# Editorials

**Published:** 1911-12

**Authors:** 


### EDITORIALS

The Business Office of the *Journal-Record* is 1 1-2 to 5 1-2 South Broad Street.
The Editorial Office is 1014-15 Century Building.
Address all Business Communications to *Journal-Record of Medicine*, 1 1-2 to 5 1-2 South Broad Street.
Make remittances payable *to The Journal-Record of Medicine.*

On matters pertaining to the Editorial and Original Communications, address Edgar G. Ballenger, M. D., Atlanta, Ga.
Reprints of Original Articles will be furnished at cost price. Requests for the same should always be made in THE MANUSCRIPT.

We will present, postpaid, on request, to each contributor of an original article, twenty (20) marked copies of *The Journal-Record of Medicine* containing such article.

### PREVENTABLE BLINDNESS.

Cheatham, in the Kentucky Medical Journal, has called attention to the importance of the subject of preventable blindness. Lamentable as is inevitable loss of sight, still worse is the blindness which, with a little knowledge and a little care could have been prevented. It is generally estimated that 40 per cent. of bindness is caused by gonococcal inflammations of the conjunctiva. While this fast is known to the majority of physicians, midwives and the layity are almost criminally ignorant of ophthalmia neonatorium. The treatment of this disease is important, but not so important as its prevention by Crede's method.

Trachoma is another frequent cause of blindness. It is stated that 75 per cent. of untreated trachoma results in blindness. As its prevention is easier than its cure, patients and friends should be carefully warned as to the danger.

---

### THE COMING MEETING OF THE CHATTAHOOCHEE VALLEY MEDICAL ASSOCIATION.

The Chattahoochee Valley Medical and Surgical Association is to meet in La Grange, Ga., Tuesday and Wednesday, Jan. 16-17, 1912. The program shows a good number of excellent papers scheduled and undoubtedly this will prove itself to be the best attended meeting that this progressive and wide-awake Association had ever had. The railway schedules afford easy access to La Grange and there should be no excuse for our not making this a banner meeting. Especially should Atlanta men be present as very little time need be consumed on the trains—there having been few meeting places so accessible. Atlanta, too, should make an effort to secure the next meeting of this Association. We have partaken of their hospitality in many cities and towns along the Chattahoochee Valley and it seems to the writer that it is now clearly "up to us" to make an effort to bring the next meeting to Atlanta and exend to these cordial Southern physicians the hospitality they have so graciously accorded to us on many previous occasions.

Take my word for it, the meeting will be worth attending—in fact, you cannot afford to miss it.

